# Evaluation of real-world treatment outcomes in patients with distant metastatic Merkel cell carcinoma following second-line chemotherapy in Europe

**DOI:** 10.18632/oncotarget.19218

**Published:** 2017-07-13

**Authors:** Jürgen C. Becker, Eva Lorenz, Selma Ugurel, Thomas K. Eigentler, Felix Kiecker, Claudia Pföhler, Ivonne Kellner, Friedegund Meier, Katharina Kähler, Peter Mohr, Carola Berking, Gabriele Haas, Christoph Helwig, Dina Oksen, Dirk Schadendorf, Lisa Mahnke, Murtuza Bharmal

**Affiliations:** ^1^ Translational Skin Cancer Research (TSCR), German Cancer Research Center (DFKZ) Partner Site Essen/Düsseldorf, Essen University Hospital, 45147, Essen, Germany; ^2^ Department of Dermatology, University Hospital of Essen, 45122 Essen, Germany; ^3^ IMS HEALTH GmbH and Co OHG, 60598, Frankfurt am Main, Germany; ^4^ Department of Dermatology, University Hospital of Tübingen, 72076 Tübingen, Germany; ^5^ Charité Universitätsmedizin Berlin, Department of Dermatology, 10117 Berlin, Germany; ^6^ Saarland University Medical School, Department of Dermatology, 66421 Homburg/Saar, Germany; ^7^ Helios-Klinik, Department of Dermatology, 99089 Erfurt, Germany; ^8^ Skin Cancer Center, University Cancer Centre, and National Center for Tumor Diseases Dresden, 01307 Dresden, Germany; ^9^ Universitätsklinikum, Department of Dermatology, 24105 Kiel, Germany; ^10^ Elbe-Kliniken, Skin Cancer Center, 21614 Buxtehude, Germany; ^11^ University Hospital Munich (LMU), Department of Dermatology and Allergy, 80337 Munich, Germany; ^12^ Merck KGaA, 64293 Darmstadt, Germany; ^13^ EMD Serono, Inc., Billerica, MA, 01821, USA; ^14^ Present address: Institute for Medical Statistics, Epidemiology and Informatics, University Medical Center Mainz, 55131 Mainz, Germany; ^15^ Department of Dermatology, University Hospital of Würzburg, 97080, Würzburg, Germany; ^16^ Department of Dermatology, University Hospital Carl Gustav Carus at the TU Dresden, 01307 Dresden, Germany

**Keywords:** Merkel cell carcinoma, skin cancer, retrospective study, chemotherapy, observational study

## Abstract

**Background and aims:**

Merkel cell carcinoma (MCC) is a rare, aggressive skin cancer; few treatments exist for patients with advanced disease. Once tumors metastasize to distant sites, patients generally receive chemotherapy, but response duration and progression-free survival (PFS) are typically short. Few studies have assessed the efficacy of second-line chemotherapy for metastatic MCC. Here, we studied outcomes in patients who received ≥ 2 lines of chemotherapy for metastatic MCC.

**Materials and methods:**

Patients in an MCC-specific registry diagnosed with stage IV MCC between November 1, 2004, and September 15, 2015, and treated with second-line or later chemotherapy were analyzed retrospectively. Patient records, including baseline characteristics, immunocompetent status, and responses to prior chemotherapy, were evaluated. Patients meeting eligibility criteria were followed through December 31, 2015.

**Results:**

Of 29 patients with metastatic MCC and immunocompetent status who had received ≥ 2 lines of chemotherapy, 3 achieved a partial response, for an objective response rate (ORR) of 10.3% (95% CI, 2.2–27.4). In the overall population including patients with immunocompetent and immunocompromised status (*n* = 34), the ORR was 8.8% (95% CI, 1.9–23.7). The median duration of response was 1.9 months (range, 1.3–2.1 months; 95% CI, 1.3–2.1). In the immunocompetent population, median PFS and overall survival were 3.0 months (95% CI, 2.5–6.0) and 5.3 months (95% CI, 4.3–6.0), respectively.

**Conclusions:**

The low response rates and limited durability confirm previous reports of the ineffectiveness of second-line or later chemotherapy in patients with metastatic MCC and provide a benchmark for assessing clinical benefit of new treatments.

## INTRODUCTION

Merkel cell carcinoma (MCC) is a rare, aggressive skin cancer that is more prevalent in elderly and immunocompromised patients [1, 2]. MCC is associated with Merkel cell polyomavirus in approximately 80% of cases [3], although tumorigenesis can also be linked to ultraviolet radiation-induced DNA damage [4–6]. MCC generally presents with lesions that are clinically unremarkable in appearance and are most commonly found on the head and neck regions and subsequently undergo rapid growth [2, 7]. The immune status of the patient is the most reliable independent predictor of survival, highlighting the role that the immune system plays in controlling malignant growth in MCC [8, 9]; specifically, high levels of intratumoral CD8+ T cells are associated with longer survival [8]. Further evidence of immune involvement in MCC comes from cases of nodal disease found in the absence of a primary tumor; this suggests that cell-mediated responses may be able to clear primary tumors in some patients [10, 11]. Additionally, overall survival (OS) rates are higher in patients with unknown primary tumors than in patients with known primary tumors. Reflecting the survival benefit observed in these patients, occult nodal disease and clinically detected nodal disease with unknown primary tumor were classified as stage IIIA in the most recent American Joint Committee on Cancer staging (AJCC) system update [10].

MCC is associated with a poor prognosis. MCC-specific 5-year survival rates reported in patients with distant metastatic (stage IV) disease, defined by metastasis beyond regional lymph nodes, range from 0% to 18% [1, 11]. The mortality rate of MCC exceeds that of other, more common skin cancers, such as melanoma [12]. In patients diagnosed with local or regional disease, the reported rate of disease recurrence was as high as 43%–48% [11].

Recent FDA approval of avelumab represents the first and only approved treatment option for metastatic MCC [13]. Historically, there have been no approved or evidence-based standard treatments for metastatic MCC and standard chemotherapy regimens for metastatic MCC include carboplatin or cisplatin with etoposide and topotecan [14, 15]. Although MCC is sensitive to these chemotherapy regimens, responses are not durable and are often associated with high toxicity in elderly patients [14, 15]. Retrospective studies have shown that response rates to first-line chemotherapy range from 52% to 61% in the distant metastatic setting [16–21], and response duration ranges from 3 to 10 months. Progression-free survival (PFS) and OS are typically measured in months [16–21]. Data for responses to second-line or later treatment are very limited, with only one full report of patients with distant metastatic (stage IV) disease published in the literature to date [20]. In this study population (*n* = 30), the objective response rate (ORR) was 23%, median duration of response (DoR) was 3.3 months, and median PFS was 2 months.

Currently, there are no prospective studies of outcomes following second-line treatment of distant metastatic MCC in European patients. Because of the rare and aggressive nature of metastatic MCC, the lack of benefit with standard chemotherapy treatments, and the emergence of promising new treatment options [22–24], it is unlikely that large prospective clinical trials with comparator chemotherapy arms will be performed [25]. To interpret the outcomes reported in recent clinical trials of immunotherapy for patients with metastatic MCC [22–24], it is necessary to evaluate the clinical activity of chemotherapy through retrospective analysis of real-world data. Here, we present the results of an observational real-world–data study designed to analyze outcomes in a European patient population with distant stage IV metastatic MCC who received second-line or later chemotherapy. Notably, to the best of our knowledge, this is the largest retrospective series on second-line chemotherapy in stage IV MCC. The patients analyzed represent those with the most advanced and difficult-to-treat MCC disease.

## MATERIALS AND METHODS

### Inclusion and exclusion criteria

Patients included in this analysis were adults aged ≥ 18 years diagnosed with distant metastatic MCC and treated with ≥ 2 lines of systemic chemotherapy for metastatic disease. Patients were excluded if they had a history of any other solid tumor within 3 years before the start of treatment for MCC, except for basal or squamous cell carcinoma, bladder carcinoma *in situ*, or cervical carcinoma *in situ*. Patients with immunocompromised status due to specific hematologic diseases (chronic lymphocytic leukemia, multiple myeloma, or hypogammaglobulinemia) or immunosuppressive treatments were eligible, although the main analysis included only immunocompetent patients. Other criteria suggestive of immunocompromised status, such as organ transplant or HIV infection, were not recorded in the MCC registry and thus not available as a screening factor in this analysis.

### Data collection

Retrospective anonymized patient-level information was extracted from an observational, real-world MCC-specific registry that was established in 2005 in German-speaking countries. Patients were identified through a collaboration between IMS Health and the German Cancer Research Center (Deutsches Krebsforschungszentrum). Data in the registry were collected from 56 clinical sites (53 in Germany, 2 in Austria, and 1 in Switzerland), including data on demographics, medical history of skin cancer and immunosuppression, clinical characteristics, treatment, and outcomes. Informed consent was given by all patients who enrolled in the MCC registry. Records from November 1, 2004, through September 15, 2015, were searched, and qualifying patients were followed through December 31, 2015.

### Outcome measures and statistical considerations

Best overall response (BOR) was assigned to each patient based on clinical judgment by the reporting physician. Because reporting according to Response Evaluation Criteria In Solid Tumors (RECIST) [26, 27] was not standard clinical practice in the countries of the registry, confirmation of response or stable disease was based on follow-up radiological imaging procedures. In case of visible disease progression, physician evaluation of clinical appearance was used and additional imaging was performed only if needed for therapeutic decisions. ORR was calculated as the proportion of patients who had a complete or partial response. Median duration of treatment was reported separately for each line of chemotherapy received, whereas time to treatment discontinuation (TTD) was reported jointly for second-line and third-line chemotherapy. Kaplan-Meier estimates were used for all time-to-event analyses. Durable response rate (DRR) was calculated as the proportion of patients who had a complete or partial response lasting ≥ 6 months. Positive visceral metastasis status was defined as the presence of metastases to sites beyond lymph nodes, skin, and soft tissue and/or elevated lactate dehydrogenase according to classification of malignant melanoma [28].

### Study objectives

The primary objective was to determine the ORR achieved with second-line or later chemotherapy in immunocompetent patients. Secondary objectives included assessment of DoR, PFS, OS, and DRR. Time to progression (TTP) was also analyzed for patients who had disease recurrence or progression. Safety was not assessed in this study. All study objectives were analyzed in the main (immunocompetent) and overall (immunocompetent plus immunocompromised meeting eligibility criteria) populations. Responses to prior first-line chemotherapy were also recorded.

## RESULTS

### Patient population

**Figure 1:**
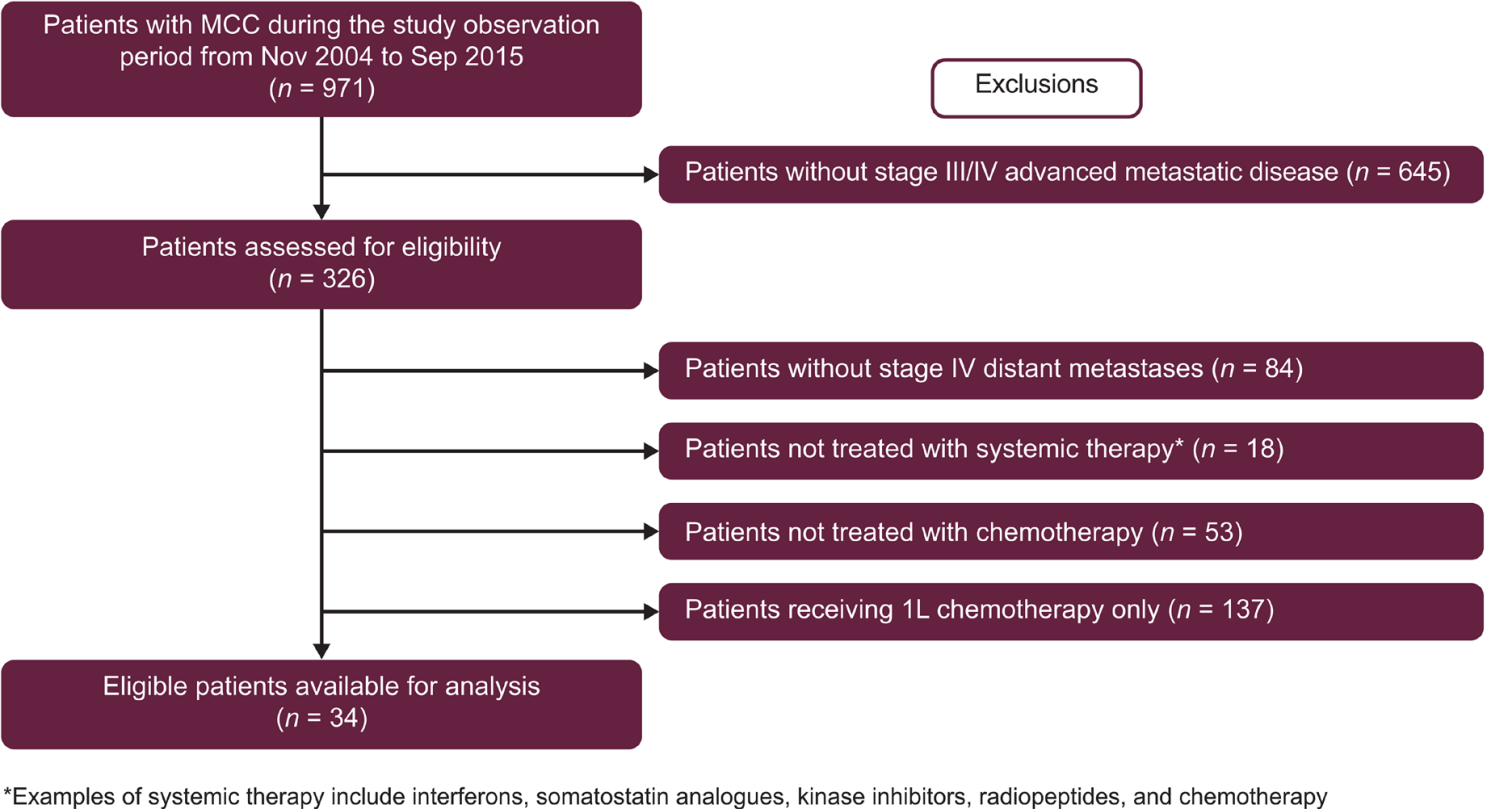
Patient selection 1L, first-line; MCC, Merkel cell carcinoma.

Data from 971 patients with MCC registered between November 01, 2004, and September 15, 2015, were available for analysis (Figure 1). Of these patients, 242 (24.9%) had been diagnosed with stage IV disease, including 171 (17.6%) who had stage IV disease treated with systemic chemotherapy, and 34 (3.5%) who had also received ≥ 2 prior lines of chemotherapy. The main analysis population comprised 29 patients classified as immunocompetent. Five patients classified as immunocompromised were included in an analysis of the overall second-line or later population (*n* = 34). Two patients were excluded from the analysis of responses to first-line chemotherapy because distant metastatic MCC had not been diagnosed when their first-line therapy was initiated. These patients did qualify for analysis of outcomes with second-line or later treatment because the requirement for any chemotherapy for MCC in the first-line setting was met.

### Baseline characteristics and treatment

In the main analysis population (immunocompetent patients), median age was 67 years (range, 36–80 years), and 62.1% of patients were male (Table 1). Primary lesions occurred mainly on the scalp or neck (20.7%) and extremities (44.8%), with 1 case of unknown primary tumor (3.5%). Most patients had stage III (48.3%) or stage IV (24.1%) disease at the time of initial diagnosis. All 34 patients had received ≥ 2 lines of chemotherapy and 5 patients, all of whom were immunocompetent, had received third-line treatment. At the initiation of first-line and second-line therapy, visceral metastasis was evident in 37.9% and 55.2% of patients, respectively.

**Table 1:** Patient and disease characteristics at baseline

	Immunocompetent (*n* = 29)	Overall (*n* = 34)
Sex, *n* (%) Male Female	18 (62.1) 11 (37.9)	22 (64.7) 12 (35.3)
Age group, *n* (%) < 55 years 55 –< 65 years 65 –< 75 years ≥ 75 years Median age (range), years	6 (20.7) 5 (17.2) 13 (44.8) 5 (17.2) 67.0 (36–80)	7 (20.6) 5 (14.7) 17 (50.0) 5 (14.7) 67.5 (36–80)
Stage at diagnosis, *n* (%) IA IB IIA IIB IIIA IIIB IV	1 (3.5) 2 (6.9) 3 (10.3) 2 (6.9 10 (34.5) 4 (13.8) 7 (24.1)	1 (2.9) 2 (5.9) 3 (8.8) 2 (5.9) 10 (29.4) 6 (17.7) 10 (29.4)
Primary tumor location, *n* (%) Arm Scalp and neck Trunk Leg Unknown primary Missing	9 (31.0) 6 (20.7) 5 (17.2) 4 (13.8) 1 (3.5) 4 (13.8)	9 (26.5) 8 (23.5) 6 (17.7) 5 (14.7) 1 (2.9) 5 (14.7)
Other skin cancer history, *n* (%) None Squamous cell carcinoma Basal cell carcinoma	24 (82.8) 4 (13.8) 1 (3.5)	29 (85.3) 4 (11.8) 1 (2.9)
Prior lines of chemotherapy for distant metastatic disease, *n* (%) 1^a^ 2 3	28 (96.6) 29 (100) 5 (17.2)	32 (94.1) 34 (100) 5 (14.7)

^a^Two patients were excluded from the analysis of responses to first-line chemotherapy due to lack of confirmed distant metastatic Merkel cell carcinoma at the time of first-line therapy initiation.

Baseline patient and disease characteristics were similar in the main (immunocompetent) and overall (immunocompetent and immunocompromised) populations. Of the 5 immunocompromised patients, 4 had B-cell chronic lymphocytic leukemia and 1 had received immunosuppressive treatment, and all 5 patients had visceral metastases at initiation of first-line therapy. There was no association between immunocompromised status and a history of other non-melanoma skin cancers.

Among patients in the main analysis population who had received at least second-line chemotherapy, the median treatment duration was 4.5 months (range, 1.8–6.0 months) with first-line chemotherapy, 2.6 months (range, 1.5–5.9 months) with second-line chemotherapy, and 2.5 months (range, 1.6–3.2 months) with third-line chemotherapy. All patients had discontinued first-line treatment because of disease progression. Second-line treatment was discontinued because of disease progression (93.1%) or death (6.9%).

Chemotherapy regimens for MCC across all treatment lines are presented in Table 2. The most common prior first-line regimens in patients classified as immunocompetent were paclitaxel (34.5%) and liposomal doxorubicin/doxorubicin monotherapy (31.0%). Among second-line therapies, doxorubicin monotherapy was the most common (34.5%), followed by carboplatin in combination with etoposide (27.6%) then paclitaxel monotherapy (13.8%).

**Table 2:** Chemotherapy regimens and treatment duration in different lines of therapy

	Immunocompetent (*n* = 29)		Overall (*n* = 34)	
	*n*	%	*n*	%
First-line regimens Liposomal doxorubicin Carboplatin + etoposide Carboplatin + paclitaxel Cisplatin + etoposide Cisplatin + paclitaxel Cyclophosphamide + methotrexate + 5-fluorouracil Doxorubicin Etoposide Paclitaxel	8 1 1 4 2 1 1 1 10	27.6 3.5 3.5 13.8 6.9 3.5 3.5 3.5 34.5	10 1 1 6 2 1 1 1 11	29.4 2.9 2.9 17.7 5.9 2.9 2.9 2.9 32.4
Second-line regimens Carboplatin + etoposide Carboplatin + paclitaxel Cisplatin + etoposide Cisplatin + paclitaxel Cyclophosphamide + doxorubicin + vincristine Doxorubicin Liposomal doxorubicin Paclitaxel	8 1 3 1 2 3 7 4	27.6 3.5 10.3 3.5 6.9 10.3 24.1 13.8	9 2 3 1 2 3 10 4	26.5 5.9 8.8 2.9 5.9 8.8 29.4 11.8
Third-line regimens Cisplatin + etoposide Doxorubicin Etoposide Paclitaxel Temozolomide	1 1 1 1 1	20.0 20.0 20.0 20.0 20.0	1 1 1 1 1	20.0 20.0 20.0 20.0 20.0
	Median	Range	Median	Range
Duration of treatment, months First-line Second-line Third-line	4.5 2.6 2.5	1.8–6.0 1.5–5.9 1.6–3.2	4.6 2.6 2.5	1.7–6.0 1.4–5.9 1.6–3.2

### Response to second-line or later chemotherapy

No patient had a complete response to second-line chemotherapy, whereas 3 patients (all immunocompetent) had a partial response, resulting in an ORR of 10.3% (95% CI, 2.2–27.4) in the main analysis population (Table 3). All 5 patients who were classified as immunocompromised had progressive disease as their BOR.

**Table 3:** Summary of responses to second-line or later chemotherapy

	Immunocompetent (*n* = 29)	Overall (*n* = 34)
Complete response, *n* (%)	0	0
Partial response, *n* (%)	3 (10.3)	3 (8.8)
Stable disease, *n* (%)	3 (10.3)	3 (8.8)
Progressive disease, *n* (%)	23 (79.3)	28 (82.4)
ORR (95% CI), %	10.3 (2.2–27.4)	8.8 (1.9–23.7)
Median DoR (range [95% CI]), months	1.9 (1.3–2.1 [1.3–2.1])	1.9 (1.3–2.1 [1.3–2.1])
DRR (95% CI), %	0.0 (0.0–11.9)	0.0 (0.0–10.3)
Median TTD (95% CI), months	2.8 (2.5–4.3)	2.7 (2.5–2.9)

DoR, duration of response; DRR, durable response rate; ORR, overall response rate; TTD, time to treatment discontinuation.

Median TTP for all patients based on Kaplan-Meier estimate was 3.0 months. In immunocompetent patients with a BOR of partial response, stable disease, or progressive disease, median TTP was 5.8, 4.6, and 2.9 months, respectively. No patients were censored for analysis because all patients died within the study period.

**Figure 2:**
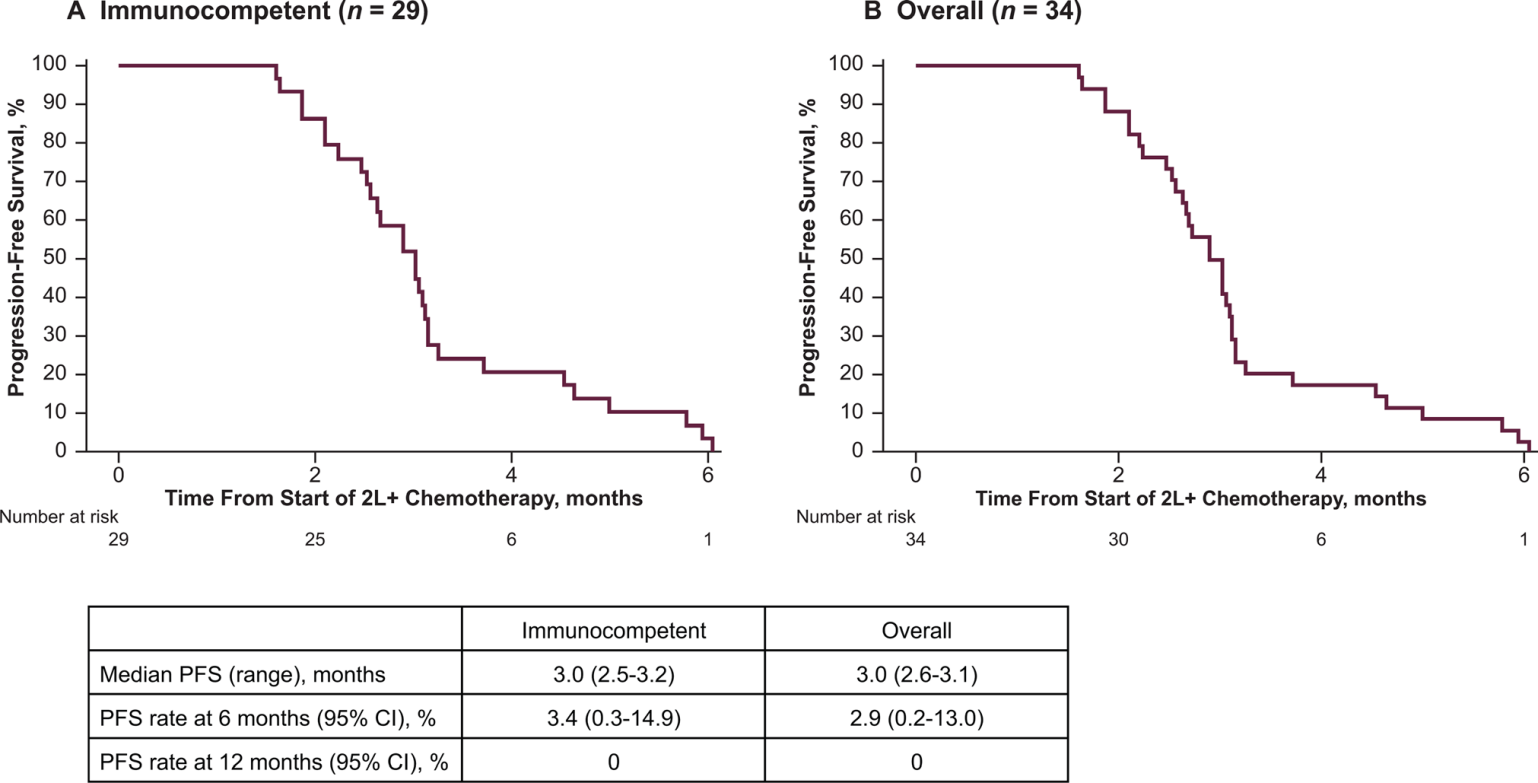
Progression-free survival (PFS) following second-line or later (2L+) chemotherapy

**Figure 3:**
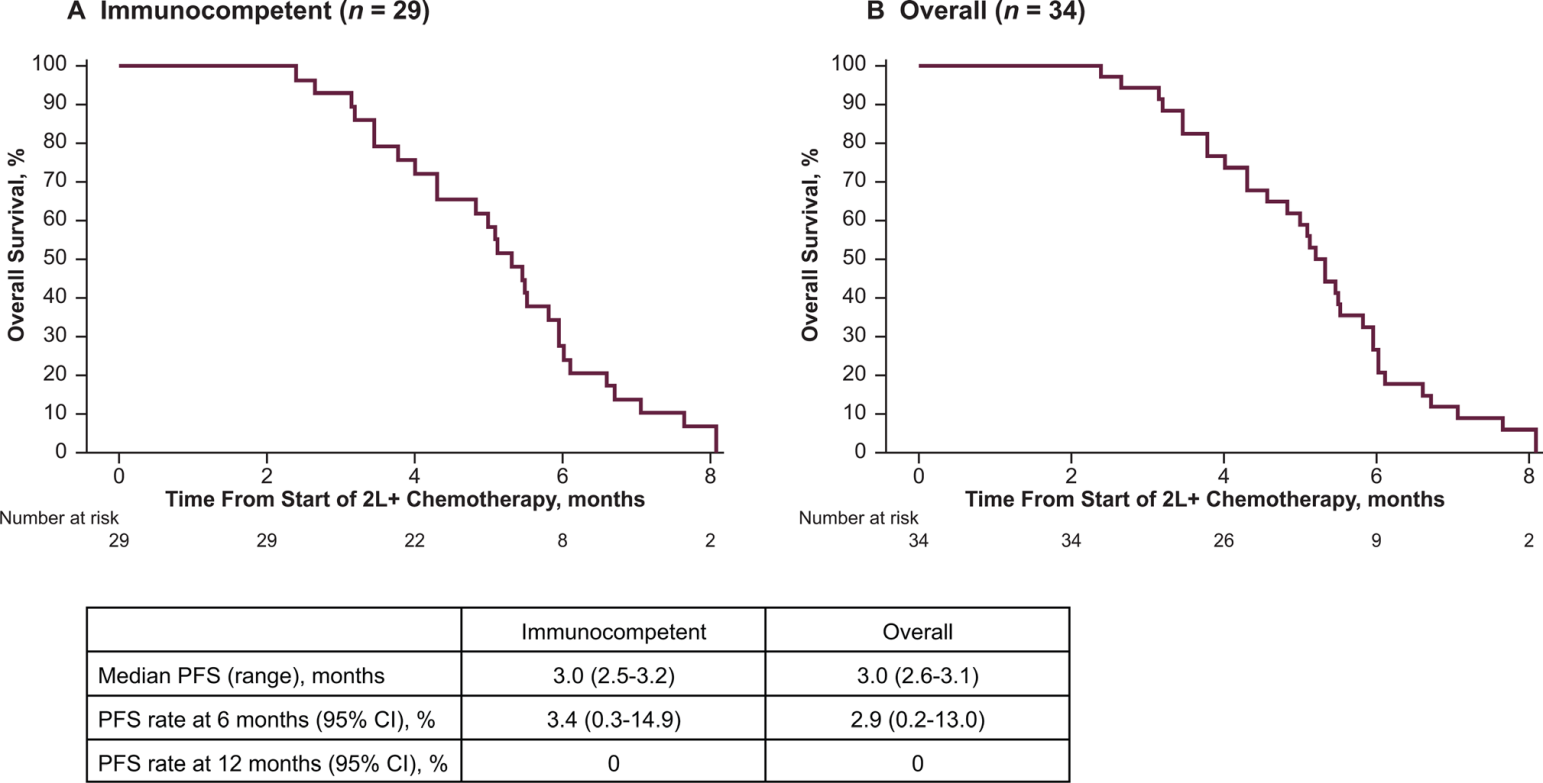
Overall survival (OS) following second-line or later (2L+) chemotherapy

Responses to chemotherapy were of limited duration (Table 3). Median DoR was 1.9 months (range, 1.3–2.1 months; 95% CI, 1.3–2.1), and because no response lasted for 6 months, the 6-month DRR was 0% (95% CI, 0.0–11.9). Median TTD was 2.8 months (95% CI, 2.5–4.3). In the main analysis population, median PFS was 3.0 months (95% CI, 2.5–3.2; Figure 2) and median OS was 5.3 months (95% CI, 4.3–6.0; Figure 3). PFS rates at 6 and 12 months were 3.4% (95% CI, 0.3–14.9), and 0%. OS rates at 6 and 12 months were 27.5% (95% CI, 13.0–44.2) and 0%. The PFS and OS data were not censored, as all patients on this study had disease progression or died.

### Response to first-line chemotherapy in patients with distant metastatic MCC who subsequently received second-line treatment

Patient outcomes with first-line chemotherapy were analyzed in 32 patients, of whom 28 (87.5%) were classified as immunocompetent and qualified for inclusion in the main analysis group (Table 4). No patient had a complete response to first-line treatment whereas 13 patients (46.4%) in the main analysis group had a partial response, resulting in an ORR of 46.4% (95% CI, 27.5–66.1). Of the 5 immunocompromised patients, 1 had a partial response. In the main analysis, median DoR was 3.3 months (range, 2.1–6.4; 95% CI, 2.4–3.7), median TTD was 4.5 months (95% CI, 2.9–5.2), and the DRR was 3.6% (95% CI, 0.1–18.3). Six months after first-line treatment was initiated, the PFS rate was 17.9% (95% CI, 6.5–33.7) and the OS rate was 96.4% (95% CI, 77.2–99.5). PFS and OS rates at 12 months were 0% and 28.6% (95% CI, 13.5–45.6), respectively.

**Table 4:** Summary of responses to first-line chemotherapy

	Immunocompetent (*n* = 28)	Overall (*n* = 32)
Complete response, *n* (%)	0	0
Partial response, *n* (%)	13 (46.4)	14 (43.8)
Stable disease, *n* (%)	4 (14.3)	5 (15.6)
Progressive disease, *n* (%)	11 (39.3)	13 (40.6)
ORR (95% CI), %	46.4 (27.5–66.1)	43.8 (26.4–62.3)
Median DoR (range [95% CI]), months	3.3 (2.1–6.4 [2.4–3.7])	3.1 (2.1–6.4 [2.4–3.7])
DRR (95% CI), %	3.6 (0.1–18.3)	3.1 (0.1–16.2)
Median TTD (95% CI), months	4.5 (2.9–5.2)	4.6 (2.9–4.8)

DoR, duration of response; DRR, durable response rate; ORR, overall response rate; TTD, time to treatment discontinuation.

## DISCUSSION

This observational, real-world-data study investigated the efficacy of chemotherapy in patients with distant metastatic MCC. Although prospective trials of chemotherapy have not been conducted, retrospective and real-world-data analyses of heterogeneous advanced disease populations have suggested that MCC is a chemosensitive malignancy [17–20]. In this analysis of immunocompetent patients who had received at least one prior line of chemotherapy in the distant metastatic setting, the ORR for the current (second- or later) line was 10.3% (partial response in 3 of 29 patients); furthermore, no immunocompromised patients responded to second-line or later chemotherapy. Median DoR was 1.9 months (range, 1.3–2.1 months; 95% CI, 1.3–2.1). Median PFS and OS were 3.0 months (95% CI, 2.5–3.2) and 5.3 months (95% CI, 4.3–6.0), respectively. While the low number of patients (*n* = 34) eligible for this study may limit the confidence in response evaluation, this study represents a carefully selected group of patients with distant metastatic MCC and allows for indirect comparisons with modern clinical studies in metastatic MCC. Additionally, this study represents the largest retrospective series reporting on outcomes of second-line or later chemotherapy.

Patients in this study were also analyzed for response to first-line treatment administered prior to second-line chemotherapy. The ORR to first-line treatment was 46.4%, although responses were also of short duration (median 3.3 months; range, 2.1–6.4 months; 95% CI, 2.4–3.7), and the median PFS from the date of first-line treatment initiation was only 4.7 months (95% CI, 3.3–5.1). An obvious limitation of the first-line analysis is that all patients received second-line or later treatment per inclusion criteria; thus, those who were unable to receive second-line therapy after first-line therapy, eg, due to rapid deterioration, were not eligible for this study. These excluded patients may also have been less healthy in general than the patients able to receive second-line or later treatment. Thus, these findings might not be generalizable to the first-line setting for distant metastatic MCC.

A recent retrospective analysis of 30 patients with distant metastatic MCC enrolled in a US-based repository also found that responses to second-line chemotherapy were of very short duration (ORR, 23%; median DoR, 3.3 months [range, 0.2–7.4 months]; median PFS, 2.0 months) [20]. In addition, a separate US-based study of 14 immunocompetent patients with distant metastatic disease receiving second-line or later chemotherapy using real-world data from US Oncology Network (USON) practices reported an ORR of 28.6%, a median DoR of 1.7 months (95% CI, 0.5–3.0), and a median PFS of 2.2 months [29]. Because responses to chemotherapy are short-lived, it is possible that the higher ORR in the US studies compared with our findings may be due to the earlier and more frequent assessment of tumors in clinical practices in the United States. Additionally, the current study did not evaluate response based on RECIST. Response was evaluated using follow-up radiological imaging procedures according to institutional practice and response was assessed based on physician judgment. Overall, our study was consistent with the 2 US studies, emphasizing the limited benefit of second-line chemotherapy in patients with metastatic MCC.

The literature characterizing outcomes of patients treated with chemotherapy for both regional and distant metastatic disease is scant and limited to summaries of retrospective case studies or anecdotal case reports. Due to the potential for reporting bias and reliance on summary data for evaluable patients only, actual ORR and DoR data may be lower than what is reported in the literature. Provided these limitations, it can be summarized that MCC is described as a chemosensitive tumor with a short DoR, although rare cases of prolonged response duration have been reported. Current treatment guidelines similarly acknowledge evidence for chemosensitivity while also noting the lack of response durability and high toxicity in elderly patients [15, 16]. Overall, the ORR observed in the literature, based mainly on patients with stage IV MCC not previously treated, ranges from 52% to 61%, with a median DoR of 3 to 9 months [17–20]. As noted, in the single published study of patients with stage IV disease treated with chemotherapy in a second-line setting, ORR was 23% and median DoR was 3.3 months (range, 0.2–7.4) [20].

These reports, combined with this study and other recent retrospective analyses in distant metastatic MCC described above, highlight the high unmet need for effective treatment options providing durable benefit in patients with distant metastatic MCC. Because of the rarity and aggressiveness of MCC, together with a rapidly changing clinical landscape in which immune therapy is emerging [25], a large prospective clinical trial comparing novel agents with chemotherapy is not feasible. The stringent selection in our study of patients with distant metastatic MCC who had received second-line or later chemotherapy provides a benchmark to compare response rates and durability in contemporary clinical trials in this patient population. A potential limitation in comparison of this study with ongoing clinical trials is that patients with elevated (> 1) ECOG performance score, short estimated life expectancy, and concurrent renal, hepatic, and cardiovascular disease were not excluded from the study. Additionally, prior non-chemotherapy treatments (eg, radiation and surgery), dose reductions during the treatments assessed, and differences in dosing schedules between places of care were not recorded for the patients included in this study. Therefore, the effect of previous treatments on patient outcomes with chemotherapy could not be evaluated.

MCC is characterized as an immunogenic cancer based on the presence of various antigens created by viral infection or UV-induced mutations and neoantigens, which can be recognized by the immune system; furthermore, unknown primary-tumor status, assumed to be related to a prior robust antitumor immune response, is the only reliable predictor of positive outcomes in patients with MCC [3–6, 30]. MCC tumors use various mechanisms to evade the host immune response, including the upregulation of immune checkpoint proteins such as PD-L1, which suppress T-cell responses [5, 10, 31]. Recent prospective clinical trials in patients with advanced MCC have shown that anti–PD-1 or anti–PD-L1 immune checkpoint inhibitors have durable efficacy and favorable tolerability relative to chemotherapy in the first-line or second-line and later settings [22–24]. In particular, in a trial of avelumab (anti–PD-L1) performed in a stage IV metastatic MCC patient population similar to that in our study (ie, receiving second-line or later treatment), the ORR was 33% (95% CI, 23–44), with 74% of responses lasting ≥ 1 year based on Kaplan-Meier analysis, and a 1-year OS rate of 52% (95% CI, 41–62) [22, 32]. In a study of pembrolizumab (anti–PD-1) administered as a first-line treatment in patients with stage III or IV MCC, the ORR was 56% (95% CI, 35–76) and 86% of responses were ongoing at data cutoff [23]. In a study of nivolumab (anti-PD-1) as first- or second-line treatment of unresectable local and/or metastatic MCC (stage II–IV), the ORR was 64% (95% CI, 43–82) and 75% of responses were ongoing at data cutoff [24]. Median DoR was not reached in any of these studies. In contrast, the reported 6-month DRR associated with chemotherapy was 0% in our real-world study and the study by Cowey et al [29] (second-line or later chemotherapy) and 6.7% in the study by Iyer et al (second-line chemotherapy) [20]. In the absence of head-to-head trials of anti–PD-L1/PD-1 therapies vs chemotherapy, this real-world study provides an important benchmark that can inform clinical decision-making.
